# Modified One-third Tubular Plate with Hook for Distal Lateral Malleolus Fracture Fixation

**DOI:** 10.5704/MOJ.1903.013

**Published:** 2019-03

**Authors:** RY Kow, CL Low

**Affiliations:** Department of Orthopaedics, Hospital Tengku Ampuan Afzan, Kuantan, Malaysia

Dear Editor,

Fracture of the lateral malleolus is a commonly encountered problem in the orthopaedic fraternity^1^. Among the lateral malleolus fractures, Weber type B is the most common injury, accounting for 60% of all ankle fractures^2^. Anatomical reduction and fixation of the distal lateral malleolus fractures are crucial to improve the outcomes in patients^1^. They are normally done with a one-third tubular plate with or without an inter-fragmentary lag screw. One-third tubular plate is a suitable implant as there is limited soft tissue coverage overlying the lateral malleolus. A commonly encountered problem intra-operatively is limited space for distal screw placement^3^. Placement of only one distal screw is insufficient to ensure rotational stability, especially in osteoporotic bone^3^. Locking plate has been proposed to treat these fractures, but a significant increase in wound complications associated with locking compression plate discourage the use of these implants^4^.

**Fig. 1:**
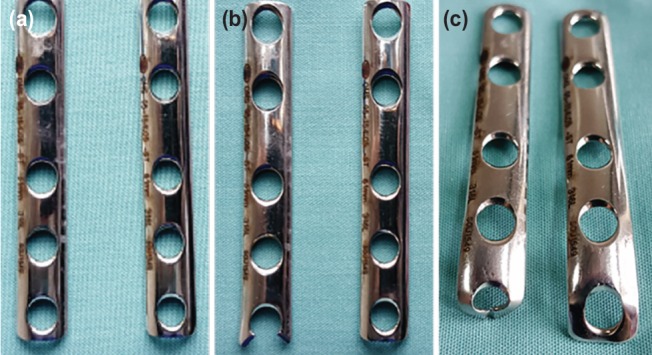
(a) Shows two similar one-third tubular plates prior to modification. (b) The distal end of the left one-third tubular plate has been cut with a plate cutter at 45 degree, creating two hooks. (c) Shows the difference between the two one-third tubular plates after bending. After cutting the plate, the end of the plate can be bent to accommodate a distal screw as well as being fitted to the distal part of the lateral malleolus. Note that the trajectory of the distal screw can now be directed at a more superior-lateral angle compared to the screw trajectory in an uncut plate.

**Fig. 2:**
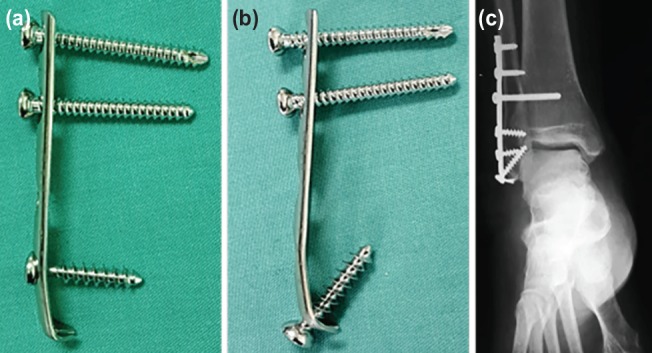
(a) The modified one-third tubular plate can be used as a hook plate with one cancellous screw being placed at the second last hole of the plate. (b) A cancellous screw directly inserted at the modified distal hole of the one-third tubular plate. (c) Ankle plain radiograph of a patient who had undergone the surgery using the described method.

Heim *et al* described a technique in which the one-third tubular plate is modified to form a hook at the distal plate. This modified implant can be used in malleolar fractures, olecranon fractures, distal radius fractures, distal ulnar fractures and proximal fractures of the fifth metatarsal^3^. Here, we would like to revisit this one-third tubular plate modified technique and to propose the use of the modified distal hole for placement of distal screw with different trajectory for better rotational stability (Fig. 1). By using this technique, the risk of ankle joint penetration by the distal screw is kept to the minimum (Fig. 2).
